# Past Obesity as well as Present Body Weight Status Is a Risk Factor for Diabetic Nephropathy

**DOI:** 10.1155/2013/590569

**Published:** 2013-08-26

**Authors:** Shu Meguro, Yusuke Kabeya, Karin Tanaka, Toshihide Kawai, Masuomi Tomita, Takeshi Katsuki, Yoichi Oikawa, Yoshihito Atsumi, Akira Shimada, Masami Tanaka, Junichiro Irie, Yoshifumi Saisho, Hiroshi Itoh

**Affiliations:** ^1^Division of Endocrinology, Metabolism and Nephrology, Department of Internal Medicine, Keio University, School of Medicine, 35 Shinanomachi, Shinjuku-ku, Tokyo 160-8582, Japan; ^2^Department of Internal Medicine, Saiseikai Central Hospital, Tokyo, Japan; ^3^Diabetes Centre, Eiju General Hospital, Tokyo, Japan

## Abstract

*Aims*. We analyzed the prevalence of nephropathy according to past body weight status in Japanese subjects with type 2 diabetes because the influence of past obesity on diabetic complications is not certain. *Methods*. We examined the prevalence of nephropathy in 2927 subjects with type 2 diabetes mellitus according to current BMI and maximum BMI in the past. We defined “current obesity” as BMI on hospitalization of 25 or more, “previous obesity” as BMI on hospitalization of less than 25 and self-reported maximum BMI in the past of 25 or more, and “continuously lean” as maximum BMI of less than 25. *Results*. The prevalence of nephropathy was significantly higher in subjects with current obesity (40.6%) or previous obesity (35.6%) than in those who were continuously lean (24.3%) (*P* < 0.017). In logistic regression analysis, previous obesity, as well as current obesity, was a significant risk factor for nephropathy, independent of sex, age, disease duration, hypertension, dyslipidemia, HbA1c, and diabetic retinopathy. *Conclusions*. Obesity in the past, as well as the present body weight status, was a risk factor for diabetic nephropathy.

## 1. Introduction

The majority of Japanese patients with type 2 diabetes mellitus are not obese, as reported by Kosaka and Ogata more than 50 years ago [1]. This is a well-known fact about Japanese type 2 diabetes mellitus [2]. Eastern Asian subjects might share common characteristics of the disease, comparable to those reported in Korean people [3]. It is, therefore, debatable whether we can apply epidemiological evidence obtained from studies of Caucasian subjects with type 2 diabetes and obesity to eastern Asian people, especially in the fields of diabetic complications, which are influenced not only by hyperglycemia but also by obesity. For example, type 2 diabetes is a relative risk factor for cardiovascular disease in Asians, as in Western societies, but the absolute risk differs greatly between these populations [4–7].

Recently, the “legacy effect” of intensive glycemic control early after the diagnosis of diabetes was advocated based on the UKPDS follow-up study [8]. However, there is no report about the legacy effect of past obesity over a lifetime on diabetic complications. Although there have been inconsistent results as to whether obesity is a risk for diabetic nephropathy [9–12], it was reported that current obesity and maximum past body mass index (BMI) were significant risk factors for diabetic nephropathy in the Japanese [12]. Although Caucasian subjects with type 2 diabetes usually maintain their body weight status during their disease course, the majority of patients of eastern Asian ethnicity begin to lose body weight from around the time of diagnosis of diabetes [3, 13]. This was easily overlooked, even if the effect of obesity in the past persisted for a long time, because they were already nonobese at the start of clinical follow-up. To clarify the influence of past obesity on diabetic complications is an important clinical concern in patients of eastern Asian ethnicity.

We therefore analyzed the difference in the prevalence of diabetic nephropathy in Japanese type 2 diabetics according to their history of body weight status to clarify the effect of past obesity on diabetic nephropathy.

## 2. Materials and Methods

We examined subjects with type 2 diabetes whose estimated glomerular filtration rate (eGFR) was 30 mL/min/1.73 m^2^ or more and who were admitted to Saiseikai Central Hospital from January 1999 to December 2004 (*n* = 1834) or Keio University Hospital from April 1998 to September 2010 (*n* = 1093) for the management of metabolic control. The study protocol was reviewed and approved by the ethics committee of both hospitals. Those subjects in whom the etiology of renal disease was strongly suspected to be other than diabetic nephropathy were excluded. According to the history of body weight status and the Japanese criteria for obesity [14], we defined “current obesity” as BMI on hospitalization of 25 or more, “previous obesity” as BMI on hospitalization of less than 25 and self-reported maximum BMI in the past of 25 or more, and “continuously lean” as maximum BMI of less than 25.

HbA1c level on admission was determined by high-performance liquid chromatography (HPLC: Arkray Inc., Kyoto, Japan) according to the recommended method by the Japan Diabetes Society (JDS) at that time and converted to the National Glycohemoglobin Standardization Program (NGSP) value [15]. eGFR (mL/min/1.73 m^2^) was calculated as 194 × Cr^−1.094^ × Age^−0.287^ (with further multiplication by 0.739 for female subjects) using the equation provided by the Japanese Society of Nephrology [16].

Subjects with albumin excretion rate (AER) of 20 *μ*g/min or more in 24-hour urine were considered to have diabetic nephropathy. All subjects underwent funduscopic examination by trained ophthalmologists during or just before admission. The diagnosis of diabetic retinopathy was made based on the Davis classification [17]. Hypertension was defined as systolic blood pressure >140 mmHg, diastolic blood pressure >90 mmHg, or the prescription of antihypertensive medication. Dyslipidemia was defined as LDL cholesterol >3.63 mmol/L, triglyceride >1.72 mmol/L, HDL cholesterol <1.04 mmol/L, or the prescription of lipid-lowering medication.

Continuous variables are expressed as mean ± SD. Differences in baseline characteristics among the obesity categories were analyzed by ANOVA and chi-squared test. Chi-squared test was also performed to evaluate differences in prevalence, with Bonferroni's correction for post hoc multiple comparisons. As a result, the probability equivalent to the usual *P* = 0.05 was *P* = 0.017. Logistic regression analysis with forced entry method was performed to detect significant independent predictors of diabetic nephropathy. We adopted as covariates factors such as sex, age, disease duration, hypertension, dyslipidemia, HbA1c, and obesity status (current obesity, past obesity, and continuously lean) in this study. Obesity status was converted to two dichotomous variables with dummy coding. *P* < 0.05 was considered statistically significant. All analyses were performed using IBM SPSS software ver. 18.0 (SPSS Inc., an IBM company, Japan).

## 3. Results

We analyzed a total of 2927 persons (males 2038, females 889, age 59.3 ± 10.6 years, BMI 24.0 ± 4.0, duration of diabetes 10.4 ± 28.0 years, HbA1c 9.3 ± 2.8%). The subjects' characteristics are shown in Table 1. The prevalence of current obesity in the total subjects was 33.6%, previous obesity 41.5%, and a continuous lean state 24.8%. The prevalence of nephropathy was significantly different among the categories, and both currently obese (40.6%) and previously obese (35.6%) patients had a significantly higher prevalence of diabetic nephropathy than that in continuously lean patients (24.3%) (*P* < 0.017). 

**Figure 1:**
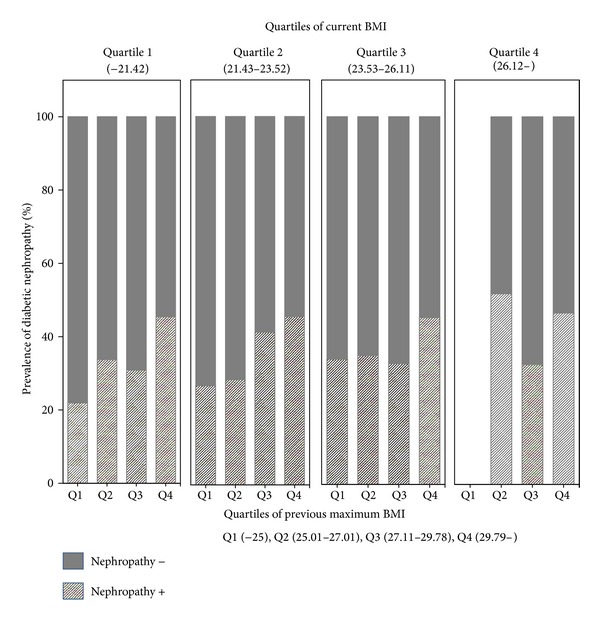
Prevalence of diabetic nephropathy divided by quartiles of current BMI and quartiles of previous maximum BMI. As for quartiles of current BMI, Quartile 1; BMI ≦ 21.42, Quartile 2; BMI: 21.43–23.52, Quartile 3; BMI: 23.53–26.11, and Quartile 4; BMI > 26.11. As for quartiles of previous maximum BMI, Quartile 1 (Q1); BMI ≦ 25.00, Quartile 2 (Q2); BMI: 25.01–27.10, Quartile 3 (Q3); BMI: 27.11–29.78, and Quartile 4 (Q4); BMI > 29.78. Prevalence of diabetic nephropathy was significantly different among the quartile groups of current BMI (*P* < 0.001 by chi-squared analysis). Prevalence of diabetic nephropathy was significantly different among the quartile groups of previous maximum BMI (*P* < 0.001 by chi-squared analysis).

When we divided the patients into quartiles according to current BMI, the prevalence of nephropathy significantly increased as current BMI increased (Figure 1, *P* < 0.001). When we similarly divided them into quartiles according to previous maximum BMI, the prevalence of nephropathy significantly increased as previous maximum BMI increased (Figure 1, *P* < 0.001). Current BMI and previous maximum BMI were highly correlated with each other (*r* = 0.785, *P* < 0.001). 

In logistic regression analysis, both current obesity and previous obesity revealed a significant odds ratio for nephropathy, as well as diabetic retinopathy, independent of sex, age, disease duration, hypertension, dyslipidemia, and HbA1c (Table 2).

## 4. Discussion 

We confirmed that previous obesity, as well as present obesity, was closely associated with nephropathy in type 2 diabetes. The notable finding of this study was that obesity is an independent risk factor, not only if it is present, but also if it was present in the past. This might indicate a legacy effect of obesity on nephropathy. The mechanism is the theme for investigation of how obesity in the past can influence diabetic complications over time.

Both current BMI and previous maximum BMI were associated with the nephropathy, as demonstrated in Figure 1. However, current BMI and previous maximum BMI were highly correlated with each other. So, generally, the higher the previous BMI, the higher the current BMI. When we analyzed the effects of previous obesity, we had to separate the effects of current obesity from those of previous obesity. This was the reason we analyzed the effect of previous obesity according to the categories defined as previous maximum BMI and current BMI. As a result, we could elucidate the effect of previous obesity on diabetic nephropathy.

Whether nephropathy in type 2 diabetes really derives from diabetes has always been a point of discussion. However, type 2 diabetes *per se* is a disease so closely linked with obesity and the metabolic syndrome that we cannot strictly distinguish the cause among the components of the syndrome. We here found that the effect of obesity was independent of the existence of diabetic retinopathy, and we expect this result can be applied to all type 2 diabetes patients. 

Several groups, including us, have reported that albuminuria was the strongest predictor of the progression of diabetic nephropathy [18–22]. We did not chronologically follow the decline of glomerular filtration rate (GFR) in this study. However, as we defined diabetic nephropathy by albuminuria, obesity, either current or past, might relate to the GFR decline through albuminuria.

Recently, the concept of obesity-related nephropathy has been advocated [23]. Although this concept is strictly defined with exclusion of both nephrosclerosis and diabetic nephropathy, the suspected mechanisms, including the contraction of efferent glomerular arterioles by the activated renin-angiotensin system (RAS) and glomerular hyperfiltration, as well as glomerular hypertrophy due to insulin resistance, are very similar to those of diabetic nephropathy. The border between the concepts of diabetic nephropathy and obesity-related nephropathy is unclear, and they cannot be distinguished clinically if a patient has both. Vivante et al. reported that overweight state and obesity in adolescents were associated with significantly increased risk for both diabetic and nondiabetic ESRD during a 25-year period [24]. If obesity even before the diagnosis of diabetes influences kidney function later, these concepts are continuous and indivisible. 

There are some limitations of this study. One is that this study was retrospective, based on self-reported body weight in the past. As for the other limitation, we might have to consider how they lost their body weight because the study subjects required metabolic interventions for poor glycemic status. The duration of obesity must be a factor of interest affecting the results. However, we only have the data of maximum body weight and the body weight on admission but not the duration. So the hypothesis needs to be confirmed in a future large cohort study with more detailed information. In spite of these limitations, this was an analysis of a large population over 10 years. We therefore believe it includes important suggestions on the effect of obesity on diabetic nephropathy.

## 5. Conclusion

Our study indicated that obesity in the past, as well as present obesity, was a risk factor for diabetic nephropathy. We should consider the effect of earlier obesity on diabetic nephropathy even if it has been present before the diagnosis of diabetes. 

## Figures and Tables

**Table 1:** Characteristics of study subjects.

	Total	Continuously lean	Previous obesity	Current obesity	*P* value
Age	59.3 ± 10.6	61.1 ± 9.4	60.4 ± 10.0	56.7 ± 11.6	<0.01
Sex (male/female)	2038/889	486/243	870/345	682/301	ns
BMI	24.0 ± 4.0	20.5 ± 2.1	22.6 ± 1.7	28.3 ± 3.2	<0.01
Max. BMI	27.7 ± 4.3	23.0 ± 1.7	27.5 ± 2.3	31.4 ± 4.1	<0.01
Disease duration (years)	10.4 ± 28.0	10.4 ± 8.4	12.2 ± 42.2	8.2 ± 7.8	<0.01
SBP (mmHg)	132 ± 18	131 ± 17	132 ± 18	133 ± 18	ns
DBP (mmHg)	75 ± 11	74 ± 11	75 ± 11	75 ± 10	ns
FPG (mmol/L)	9.9 ± 4.0	9.5 ± 4.0	9.9 ± 4.1	10.2 ± 3.9	ns
HbA1c (%)	9.3 ± 2.8	9.1 ± 2.7	9.5 ± 3.4	9.3 ± 2.0	<0.05
TC (mmol/L)	5.3 ± 1.2	5.3 ± 1.0	5.2 ± 1.0	5.5 ± 1.6	<0.01
HDL (mmol/L)	1.3 ± 0.5	1.5 ± 0.5	1.3 ± 0.4	1.2 ± 0.3	<0.01
TG (mmol/L)	1.6 ± 1.4	1.4 ± 0.2	1.5 ± 1.1	1.9 ± 1.6	<0.01
Cr (mmol/L)	66.3 ± 19.4	64.5 ± 16.8	66.3 ± 19.4	68.9 ± 20.3	<0.01
eGFR (mL/min/1.73 m^2^)	81.8 ± 28.8	81.4 ± 20.5	83.1 ± 31.1	80.6 ± 31.0	ns
Retinopathy (none/simple/proliferative)	2111/505/311	558/116/55	824/225/166	729/164/90	<0.01

Continuous variables are expressed as mean ± SD. Differences in baseline characteristics among the obesity categories were analyzed by ANOVA and chi-squared test. The numbers of each stage of diabetic retinopathy are noted on the row of retinopathy. SBP: systolic blood pressure, DBP: diastolic blood pressure, FPG: fasting blood glucose, TC: total cholesterol, HDL: HDL cholesterol, TG: triglyceride, ns: not significant.

**Table 2:** Logistic regression analysis with forced entry method for diabetic nephropathy.

	Odds ratio	95% confidence interval	*P* value
Age	1.011	1.002–1.019	<0.05
Disease duration	1.001	0.998–1.004	ns
Sex (male: 1, female: 0)	1.955	1.606–2.378	<0.001
Hypertension	1.232	1.023–1.484	<0.05
Dyslipidemia	1.306	1.096–1.556	<0.01
HbA1c	1.014	0.984–1.044	ns
Diabetic retinopathy	3.856	3.214–4.626	<0.001
Previous obesity: 1, other: 0	1.656	1.323–2.073	<0.001
Current obesity: 1, other: 0	2.480	1.959–3.141	<0.001

Adjusted *R* ^2^ = 0.166, *P* < 0.001. ns: not significant.
